# The Potential of *Alternaria* Toxins Production by *A*. *alternata* in Processing Tomatoes

**DOI:** 10.3390/toxins14120827

**Published:** 2022-11-24

**Authors:** Qiaomei Qin, Yingying Fan, Qinlan Jia, Shuaishuai Duan, Fengjuan Liu, Binxin Jia, Guangquan Wang, Wanhui Guo, Cheng Wang

**Affiliations:** 1 College of Life Science and Technology, Xinjiang University, Urumqi 830049, China; 2 Key Laboratory of Agro-products Quality and Safety of Xinjiang, Laboratory of Quality and Safety Risk Assessment for Agri-products (Urumqi), Institute of Quality Standards & Testing Technology for Agri-products, Xinjiang Academy of Agricultural Sciences, Urumqi 830091, China; 3 College of Biology and Geography Sciences, Yili Normal University, Yining 835000, China

**Keywords:** *Alternaria* toxins, conjugated mycotoxins, field experiment, in vivo experiment, processing tomatoes

## Abstract

As a filamentous and spoilage fungus, *Alternaria* spp. can not only infect processing tomatoes, but also produce a variety of mycotoxins which harm the health of human beings. To explore the production of *Alternaria* toxins in processing tomatoes during growth and storage, four main *Alternaria* toxins and four conjugated toxins were detected by ultrahigh-performance liquid chromatography-tandem mass spectrometry (UPLC-MS/MS) and ultra-performance liquid chromatography-ion mobility quadrupole time-of-flight mass spectrometry (UPLC-IMS QToF MS) in processing tomatoes on different days after being inoculated with *A. alternata*. The results show that the content of *Alternaria* toxins in an in vivo assay is higher than that under field conditions. Tenuazonic acid (TeA) is the predominant toxin detected in the field (205.86~41,389.19 μg/kg) and in vivo (7.64~526,986.37 μg/kg) experiments, and the second-most abundant toxin is alternariol (AOH). In addition, a small quantity of conjugated toxins, AOH-9-glucoside (AOH-9-Glc) and alternariol monomethyl ether-3-glucoside (AME-3-Glc), were screened in the in vivo experiment. This is the first time the potential of *Alternaria* toxins produced in tomatoes during the harvest period has been studied in order to provide data for the prevention and control of *Alternaria* toxins.

## 1. Introduction

Processing tomato is a cultivated type of common tomatoes that received its name from its thick skins, which is resistant to transport damage and suitable for processing. Xinjiang, the valley region of California, and the Mediterranean region of Europe are known as the three major centers of tomato cultivation and processing in the world. However, during the growth and storage processes, processing tomatoes are susceptible to attack by various pathogenic and spoilage micro-organisms, which reduce their yield and quality during the growth period [1,2]. Among these fungi, *Alternaria* spp. can infect processing tomatoes and contribute to devastating fungal diseases, such as tomato early blight and black spot disease [3]. The latter disease mainly occurs after the coloring period. *A. alternata* enter the host tissue (tomato fruit) through wounds or natural openings during the harvest or pre-harvest periods [4], lurk for several days, and then appear as black spots causing *Alternaria* rot [5]. These spots appear as sunken lesions, and are mostly observed near the blossom-end or peduncle of the fruit, leading to fruit spoilage that limits the product’s marketability, in addition to causing considerable post-harvest losses [6].

*Alternaria* spp. is not only a filamentous and spoilage fungus that survives in a wide range of temperatures and attacks a wide range of economically important plants (for example, apple [7,8], tomato [8,9], pear [7,10], wheat [11], etc.), but also produces a variety of secondary metabolites with toxic properties, named as *Alternaria* toxins [12], including alternariol (AOH), alternariol monomethyl ether (AME), tenuzonic acid (TeA), tentoxin (TEN), etc [13]. In addition, these toxins can primarily be bound to sulfates or glucosides, forming the conjugated mycotoxins present in the plant host [14]. Additionally, these conjugated mycotoxins release the free toxin after being hydrolyzed during metabolism, potentially endangering the health of human beings [15]. The research conducted on *Alternaria* toxins dates back to the 1960s–1970s, when some metabolites produced by *Alternaria* spp. Were first reported to exert toxic effects [16]. A limited in vivo study showed that TeA exerted mild toxic effects on mammalian cells [17,18]. Both AOH and AME have repeatedly been reported to possess cytotoxic and, of particular concern, genotoxic properties in micromolar concentrations [19,20]. In 2011, according to the EFSA, the tolerable upper intake level, using threshold of toxicological concern (TTC) was 2.5 ng/kg body weight (b.w.) for AOH and AME and 1500 ng/kg b.w. for TeA and TEN [13]. In 2022, the European Union (EU) issued proposal 202/553 to amend regulation (EC) No 401/2006 for the monitoring of *Alternaria* toxins in food, and set limits for the toxins present in processed tomato products, in which AOH, AME, and TeA do not exceed 10, 5, and 500 μg/kg, respectively [21].

A survey conducted in Brazil revealed that neither AME nor AOH was detected in 80 samples, but TeA was observed in 7 tomato pulp (39~111 μg/kg) and 4 tomato puree (29~76 ng/kg) samples [22]. In contrast, AOH was found at levels of 13 μg/kg with high frequency in tomato (93% of 44 samples) products, reported by Ackerman et al. [23]. Approximately 60% of Argentinian tomato pulp samples were contaminated with TeA, AME, and AOH at levels up to 4021 μg/kg (29% of 80 samples), 1734 μg/kg (26% of 80 samples), and 8756 μg/kg (6% of 80 samples), respectively [24]. A survey conducted on the Swiss market in 2010 showed that TeA was found most frequently in tomato products (81 out of 85 samples) and in the highest levels of up to 790 μg/kg, while AOH and AME were found in lower concentrations, ranging from <1 to 33 μg/kg for AOH and <5 to 9 μg/kg for AME [25]. In an expanded follow-up survey, conjugated mycotoxins AOH-3-sulfate (AOH-3-S) and AME-3-sulfate (AME-3-S) were detected in 9% and 34% of tomato sauces collected in retail markets in Austria, Croatia, and Italy, and their contents were up to 2.1 μg/kg and 17.5 μg/kg, accounting for 7~100% of their parent toxin concentrations [26]. A total of 17 *Alternaria* toxins, including AOH-3-glucoside (AOH-3-Glc), AOH-9-glucoside (AOH-9-Glc), AOH-3-S, AME-3-glucoside (AME-3-Glc), and AME-3-S, were investigated in tomato sauce, sunflower seed oil, and wheat flour, and interestingly, the results determined that concentrations of AOH-9-Glc and AME-3-S were in similar to their parent toxins in a naturally contaminated tomato sauce sample [27]. These observations highlight the importance to include *Alternaria* toxins in analytical methods for food surveillance, and due to the detection of excessive *Alternaria* toxins in most tomato products, attention should be paid to the production of *Alternaria* toxins in tomato fruits.

There are only a few studies addressing the production and transformation of mycotoxins. An experiment about the infection process of *Fusarium culmorum* in wheat spikes after spray and single spikelet inoculations was presented by Kang and Buchenauer [28], who observed that the pathogen was extended in the rachis in upward and downward directions by inter- and intra- cellular growths inside and outside of the vascular bundles of the rachis. Wang et al. [29] investigated the interaction between *Penicillium expansum* and wounded apple fruit tissues during the early stages of the infection, using a Pannoramic MIDI slide scanner to determine the key time points to collect samples for the transcriptomic analysis. In 2018, Xie et al. [30] simultaneously quantified the pathway metabolites of aflatoxin biosynthesis in culture medium and revealed the dynamic changes in the biosynthesis pathway by orbitrap fusion mass spectrometry and a D-optimal mixture design method. To date, in relation to the complex mechanisms in plants leading to various factors affecting the production of mycotoxins, different types of culture medium have been used to study the transformation and metabolism of toxins. Generally, ultra-performance liquid chromatography-high resolution mass spectrometry (UPLC-HRMS) is used to identify the metabolic intermediates of mycotoxins.

Over the years, most of the current studies on the toxicity and mechanism of *Alternaria* toxins have mainly focused on the in vivo investigations and generation mechanism, and the transformation pathway of *A. alternata* in the host has not been determined. This is mainly limited by instrument conditions and toxin standards. Many standards of *Alternaria* toxins, including conjugated toxins, are lacking developed research and availability of commercial products, which require UPLC-HRMS to perform a qualitative analysis. Therefore, in this study, the potential of *Alternaria* toxins production in processing tomatoes is evaluated via ultra-high-performance liquid chromatography–tandem mass spectrometry (UPLC-MS/MS) and ultra-performance liquid chromatography–ion mobility quadrupole time-of-flight mass spectrometry (UPLC-IMS QToF MS). Moreover, the changes in *Alternaria* toxin species and contents are compared in processing tomatoes during the growth and storage periods. This study provides the basic data for the further study of the production and metabolism of *Alternaria* toxins in processing tomatoes, which is of great significance to control the quantity of *Alternaria* toxins in tomato products.

## 2. Results

### 2.1. The Lesion Diameter Affected by A. alternata

**Figure 1:**
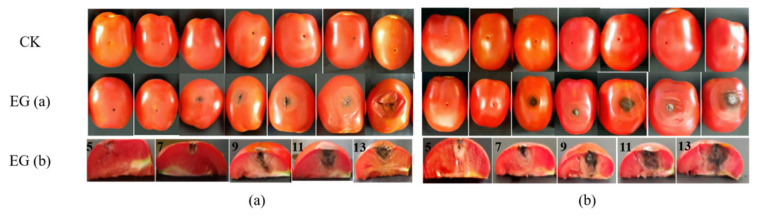
Observation of lesion diameter of tomatoes ((**a**): in field experiment; (**b**): in vivo experiment; CK: control group; EG: experimental group; from left to right in CK and EG (**a**), sunken areas can be observed on the 1st, 3rd, 5th, 7th, 9th, 11th, and 13th day post-inoculation; those in the EG (**b**) are the section plots on 5th, 7th, 9th, 11th, and 13th day post-inoculation of tomatoes).

**Figure 2:**
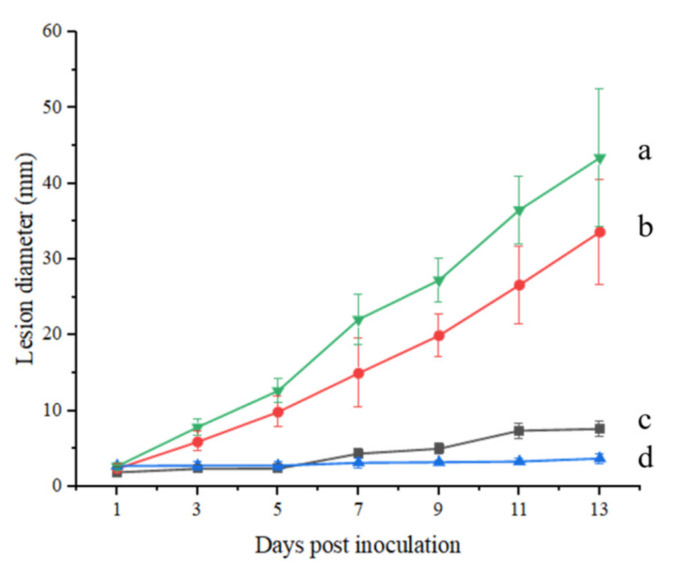
The change in lesion diameter after being inoculated (a: experimental group in in vivo experiment; b: experimental group in field experiment; c: control group in field experiment; d: control group in in vivo experiment).

The lesion diameter was measured using the cross crossing method. From Figure 1 EG (a), we can observe that the diameter of the spots in the experimental group present a significant change in the tomatoes, accompanied by the deepening of the color of the spots, while there were no changes visible in the tomatoes in the control group (Figure 1 CK). Three days post inoculation, sunken areas could be observed around the inoculation sites, and on the 5th day, the sunken areas had enlarged and turned into black lesions. In the view of the section plot (Figure 1, EG (b)), it is observed that the spread of disease spots in the in vivo experiment was more rapid than that in field experiments, and essentially penetrated the whole tomato fruit on the 11th day. Figure 2 also demonstrates that the in vivo experiment conducted on the same day appears to present slightly more plaque than the field experiment, as there is a difference in the lesion diameter (43.44 mm) relative to that of the field experiment (33.63 mm) at 13 days post-inoculation.

### 2.2. Alternaria Toxins Produced in Processing Tomatoes in the Field Experiment

#### 2.2.1. Analysis of Alternaria Toxins

**Figure 3:**
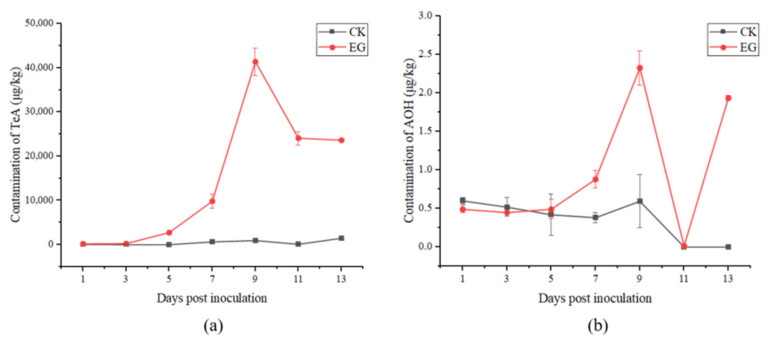
Production of tenuazonic acid (TeA) (**a**) and alternariol (AOH) (**b**) in tomatoes field experiments (CK: control group; EG: experimental group).

The production of *Alternaria* toxins in the field trials was analyzed using UPLC-MS/MS. In fact, only TeA and AOH were detected, and neither AME nor TEN was detected either in the experimental or control groups. It can clearly be observed in Figure 3 that the concentrations of all the toxins in the experimental group were almost higher than those in the control group. In the experimental group, the highest accumulation rates of TeA (41,389.19 μg/kg) and AOH (2.33 μg/kg) were presented on the 9th day post-inoculation. During the 13 days post-inoculation, the range of TeA was 205.86~41,389.19 μg/kg with the accumulation increasing from the 1st day post-inoculation to the 9th day, and then rapidly decreasing within two days. The range of AOH was <0.26~2.33 μg/kg and the change in AOH content was consistent with that of TeA, except for a sharp increase on the last two days. In the control group, the concentrations of TeA and AOH were 32.97~1494.37 μg/kg and <0.26~0.6 μg/kg, respectively, which presented a similar trend as the experimental group. TeA was the predominant toxin produced, and was significantly higher (*p* < 0.01) than the other toxins in the two groups.

#### 2.2.2. Analysis of Conjugated Alternaria Toxins

**Figure 4:**
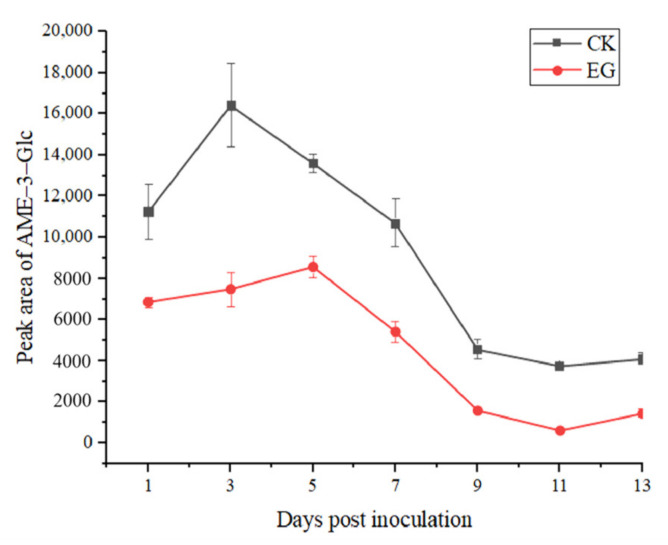
Production of alternariol monomethyl ether-3-glucoside (AME-3-Glc) in tomato field experiments (CK: control group; EG: experimental group).

As the standards of conjugated *Alternaria* toxins were not available, UPLC-IMS QToF MS was used to screen the conjugated *Alternaria* toxins, including AOH-3-S, AOH-3-S, AME-3-Glc, and AOH-9-Glc. As can be observed in Figure 4, the four conjugated toxins, except AME-3-Glc, were not detected. AME-3-Glc was detected post-inoculation, and the peak area in the control group was always larger than that in the experimental group. In the experimental group, the largest peak area of AME-3-Glc was 8579.93 on the 5th day post-inoculation, then decreased until the 11st day, and then slightly increased again. The largest peak area of AME-3-Glc in the control group was 16,412.85 on the third day post-inoculation and changed in a manner similar to that in the experimental group.

### 2.3. Alternaria Toxins Produced in Processing Tomato in In Vivo Experiment

#### 2.3.1. Analysis of Alternaria Toxins

**Figure 5:**
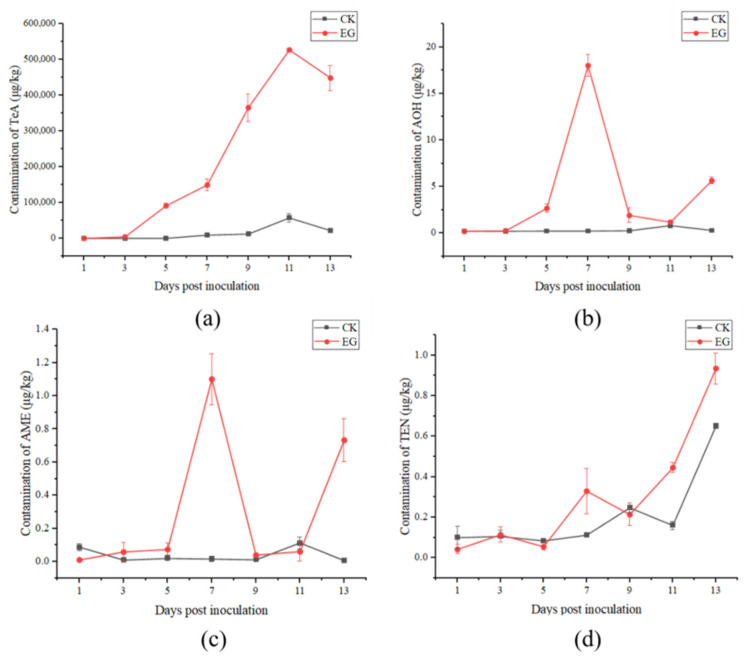
Production of TeA (**a**), AOH (**b**), alternariol monomethyl ether (AME) (**c**), and tentoxin (TEN) (**d**) in tomato in vivo experiments (CK: control group; EG: experimental group).

The results of the toxins (TeA, AOH, AME, TEN) concentrations in the tomatoes in vivo experiments are presented in Figure 5. In the experimental group, TeA continued to accumulate after inoculation, achieving a peak on the 11th day (526,986.37 μg/kg), and then it decreased. The variation trends of AOH and AME were very similar, and from the 5th day post inoculation, the toxin sharply increased, and reached the peak on the 7th day (18.02 μg/kg and 1.10 μg/kg) then sharply decreased and began to increase on the 11th day. TEN showed an overall upward trend, the highest toxin concentration was on the 13th day (0.94 μg/kg). In the control group, the toxin levels in the tomatoes inoculated with sterile water were much lower than those in the experimental group, but the changes in toxin levels were similar to those in the experimental group. Comparing the four *Alternaria* toxins, the concentration of TeA was much higher than that of the other toxins, followed by AOH. AME and TEN had lower concentrations, either in the experimental or control groups.

#### 2.3.2. Analysis of Conjugated Alternaria Toxins

**Figure 6:**
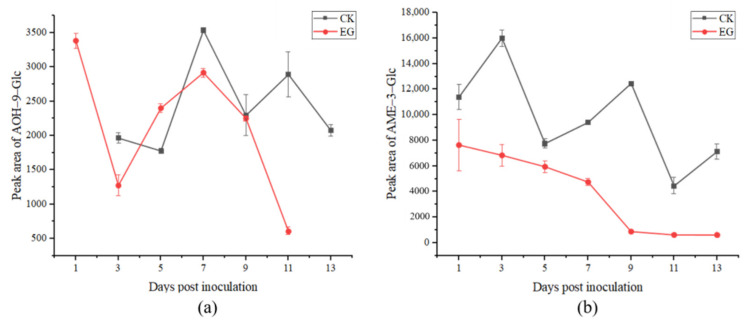
Production of alternariol-9-glucoside (AOH-9-Glc) (**a**) and AME-3-Glc (**b**) in tomato vivo experiments (CK: control group; EG: experimental group).

The results of detecting conjugated toxins in the in vivo experiment with UPLC-IMS QToF MS are presented in Figure 6. It can be observed from Figure 6a that in the experimental group, following inoculation, the highest peak area of AOH-9-Glc was detected on the 1st day (3384.52), sharply decreased on the third day, and then increased until the 7th day, and the peak area was 2918.14. In the control group, the change in AOH-9-Glc was similar to that in the experimental group, which can be clearly observed from the 5th to 13th day post-inoculation, with the highest peak area for AOH-9-Glc on the 7th day (3535.80). It can be observed in Figure 6b that the concentration of AME-3-Glc decreased until the last day post-inoculation in the experimental group, while in the control group, the peak area of AME-3-Glc present an irregular change and the highest peak area was on the 3rd day (16,006.58) post-inoculation. From Figure 6, it can be observed that the in vivo experiment, there was a downward trend of the conjugated toxins of AOH-9-Glc and AME-3-Glc as a whole.

## 3. Discussion

### 3.1. Virulence of A. alternata in the Inoculation of Tomatoes

Artificial inoculation efficiently mimics a spontaneous mold contamination. Based on the observation of tomato disease spots in the field and in vivo experiments, it was easy to observe that when the tomato was infected with *A. alternata*, it spread from the lesion center to all sides, indicating that all of the tissues in the tomato could become a medium for the growth of *A. alternata*. The change in lesions was highly consistent with black spot disease, and the research conducted by Rizwana et al. also confirmed this assessment [31].

### 3.2. Varying Potential of Alternaria Toxins Production between Field and In Vivo Experiments

In this study, we conducted field and in vivo experiments on the changes in secondary metabolites in tomatoes infected with *A. alternata*, and the wide variability of mycotoxins produced was observed in the field and in vivo experiments. Comparing the two experiments, the content of all the toxins in the in vivo experiments were more varied than that in the field experiment; a possible explanation might be that plucked tomatoes may be more susceptible to *Alternaria* infection due to senescence [32]. The great variability among *Alternaria* toxins was observed both in the field and in vivo experiments. The most frequently occurring toxin was TeA, and it was observed in significantly high levels. The range of TeA detected in the present study was 18.20~526,986.37 μg/kg, which was higher than the results for whole tomatoes (10,700~139,000 μg/kg) in Pennsylvania detected by Stinson et al. [33]. The amount of TeA was the highest in whole tomatoes and was at most four orders of magnitude higher than that of other *Alternaria* toxins, however, the highest amount of TeA was inconsistent for the variety difference of tomatoes and *Alternaria* strains. AOH and AME were produced in much lower quantities in our study, and most of the tomatoes did not contain detectable amounts of the two toxins in the other studies [33]. Sanzani et al. [34] also reported that AOH and AME were detected in few fresh tomatoes, to a lesser extent, by AME (10.2~18.3 μg/kg) and AOH (16.4 μg/kg), which had lower quantities than TeA (11~4560 μg/kg). It is understood that AOH and AME are the most important types of *Alternaria* toxins, since the toxicity of these mycotoxins can be emphasized by the combination of sulfates and glucosides produced by *Alternaria* spp. cultured on tomatoes; however, there are few data addressing the natural occurrence of AOH and AME in tomatoes in our study and other studies [35,36].

In this study, the amount of four *Alternaria* toxins in the field experiment were less than that in the in vivo experiment, but the standard deviation was higher, possibly owing to the fact that the in vivo experiment broke away from the uncertainty of the environmental factor in the field experiment. Under the conditions of appropriate temperature and water activity, the change in *Alternaria* toxin content was more obvious than that in the field experiment, but the toxin contained in the tomatoes themselves could not be excluded. The growth of and decline in *Alternatia* toxins in tomatoes can be preliminarily judged by comparing the experimental group with the control group.

### 3.3. The Production of Conjugated Mycotoxins

**Figure 7:**
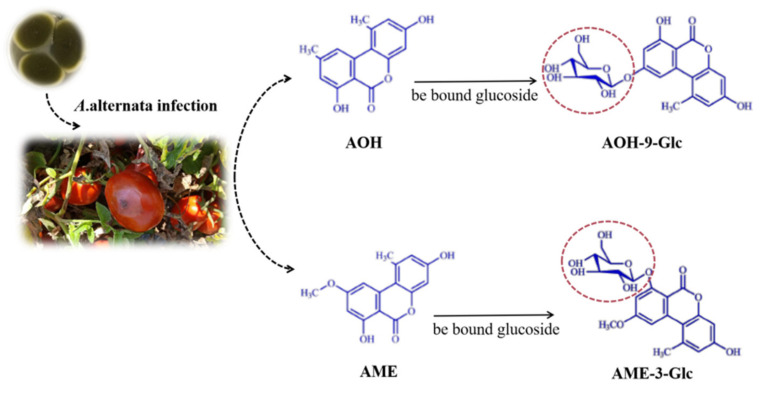
Transformation of *Alternaria* toxins in tomatoes.

In this study, AOH-9-Glc, AME-3-Glc, AOH-3-S, and AME-3-S were identified by UPLC-IMS QToF MS. As shown in Figure 7, as part of their metabolism, plants are capable of transforming phytotoxins into conjugated forms. AOH and AME can be efficiently conjugated, especially with glucose or sulfates by cultured plant cells. In this study, AOH-9-Glc and AME-3-Glc were detected alone with AOH and AME in tomatoes. It can be observed that the amount of the two conjugated toxins in the control group was higher than that in the experimental group, which is similar to the result obtained by Sebastian et al. [37], who observed that non-infected tomatoes were able to generate glucosides but not sulfates of AOH and AME, whereas *A. alternata* produced sulfates but a small quantity of glucosides. The probable reason for the low amounts of glucosides observed in the study is the fact that the analyzed specimens contained mostly fungal material and very little intact tomato tissue. Many relatively new reports suggest that conjugated mycotoxins are of relevance for exposure and risk assessments, and further studies should aim to identify additional, to-date-unknown conjugates using new technology.

## 4. Conclusions

Our results revealed a toxin production discrepancy after being infected by *A. alternata* between detached fruit and whole plant assays. In this study, four kinds of *Alternaria* toxins (AOH, AME, TeA, and TEN) and four conjugated mycotoxins (AOH-3-S, AME-3-Glc, AOH-9-Glc, and AME-3-S) were detected and investigated in processing tomatoes inoculated with *A*. *alternata* during harvest and storage periods. TeA was found to be the predominant toxin both in the field and in vivo experiments, then AOH and AME were detected in low levels. Through the screening of the conjugated mycotoxins, AME-3-Glc was detected both in the field and in vivo experiments, while AOH-9-Glc was only detected in the in vivo experiment. We propose that the content of all forms of *Alternaria* toxins detected in tomatoes may not be very accurate because the conjugated toxins can escape detection. Therefore, it is necessary to further study this more extensively to accurately understand the pollution of *Alternaria* toxins in tomatoes prior to harvesting.

## 5. Material and Methods

### 5.1. Chemical and Reagents

Anhydrous magnesium sulfate (MgSO_4_) and sodium chloride (NaCl) were purchased from Sinopharm Chemical Reagent Co., Ltd. (Shanghai, China). LC-MS grades of acetonitrile, formic acid, and acetic acid were purchased from Thermo Fisher Scientific (Waltham, MA, USA). Ultra-pure water was provided by the Watsons Group (Hong Kong) Ltd. Standard TeA, AOH, AME, and TEN were purchased from Romar Labs Division Holding GmbH (Getzersdorf, Austria), and individual standard solutions of each mycotoxin were prepared at ~100 μg/mL in acetonitrile and stored at −20 °C. Leucine enkephalin was acquired from Waters Corporation (Milford, MA, USA).

### 5.2. Instruments

The samples were weighed on an XSE 204 balance (Mettler-Toledo, Greinfesee, Switzerland), homogenized with a T13 basic ultraturrax (IKA, Staufen, Germany), and mixed in an automatic horizontal shaker (Hannuo Instruments, Shanghai, China). Centrifugation was performed using a Sorvall biofuge Stratos system (Thermo Fisher Scientific, Waltham, MA, USA).

### 5.3. Tomato, A. alternata and Spore Suspension

H1015 tomatoes were grown in an experimental field in Changji, Xinjiang Province. Sowing was performed on 1 May 2021, and a single test field that consisted of 70 to 80 6-m rows spaced 40 cm apart was used. Seeds were sown 80 cm apart in the row, resulting in a population of approximately 600 plants. *A. alternata* isolate H10, being isolated from diseased tomatoes and identified, was obtained by single-spore isolation in our laboratory and cultivated in potato dextrose agar (PDA). To obtain a spore suspension of *A. alternata* isolate H10, sterile distilled water was added to the fully overgrown PDA plates and the spore suspension was adjusted to 1 × 10^5^ conidia/mL using a hemocytometer. Suspension was used directly after preparation for the different experiments.

### 5.4. Field Experiment

The tomatoes were inoculated approximately 15 days before harvest, washed, and sanitized with 75% ethanol. Using a sterilized toothpick, uniform cuts 3 mm deep and 3 mm wide were made in both sides of the epidermis of the fruit in the equatorial region. A quantity of 15 μL of spore suspension was then inoculated into each cut, and tomatoes inoculated with sterile water were used as controls. Samples were obtained the following day after inoculation, 7 times in total.

### 5.5. In Vivo Experiment

Healthy, non inoculated, and uniform-sized tomatoes free of disease spots were selected for the experiment. After being washed with water and sanitized with 75% ethanol, using a sterilized toothpick, uniform cuts 3 mm deep and 3 mm wide were made on both side of the epidermis of the fruit in the equatorial region. A quantity of 15 μL of a spore suspension and 15 μL of sterile water were inoculated into each cut of the experimental and control groups. The fruits were stored at 25 °C (±2 °C) in sterilized, breathable boxes for 13 d, and the samples were obtained the following day after inoculation.

### 5.6. Lesion Diameter Measurement

A total of 7 samples were obtained from the experimental and control groups respectively, in both the field test and in vivo experiment, and the diameters of the spots were measured.

### 5.7. Extraction of Alternaria Toxins

The homogenized samples (5 g) were placed in 50 mL centrifugal tubes, followed by the successive addition of 10 mL of water and 10 mL of acetonitrile containing 1% acetic acid. The mixtures were shaken on an automatic horizontal shaker at 2500 rpm for 5 min to fully disperse the sample. Subsequently, 4 g anhydrous MgSO_4_ and 1 g NaCl were immediately added while vigorously shaking the tube to prevent the agglomeration of the salts. After centrifugation at 5000× *g* for 5 min, the supernatant was evaporated to near dryness (approximately 1 mL of residue remained) under a stream of nitrogen at 40 °C. Finally, 1 mL of the combined solution (acetonitrile/methanol/formic acid, 70:29:1, *v*/*v*/*v*) was added to the residue, which was vortexed, filtered through a 0.22 μm PTEE filter, and injected into the UPLC-IMS QToF MS system.

### 5.8. Alternaria Toxins Detection

The detection of *Alternaria* toxins (TeA, AOH, AME, and TEN) was performed on a Waters Acquity UPLC tandem quadrupole (TQD) mass spectrometer (Waters, Milford, MA, USA), which contained an Acquity UPLC HSS C18 (1.7 μm, 2.1 × 100 mm) column for separation. Column temperature was set at 40 °C. The mobile phase comprised methanol as eluent A and 0.1 mM ammonium carbonate as eluent B. A gradient elution was applied as follows: 20% A was initially used and linearly increased to 100% within 4 min, then maintained for 1.5 min, then decreased to 20% within 0.5 min, then maintained for 2 min, after which, column re-equilibration occurred, leading to a total run time of 8 min. The flow rate was set at 0.3 mL/min.

The MS/MS analysis was operated in the negative mode at a capillary voltage of 2.5 kV, a desolvation temperature of 600 °C, a source block temperature of 125 °C, a desolvation gas of 1000 L h^−1^, and a cone nitrogen gas flow of 150 L h^−1^. The ion chromatogram for each *Alternaria* toxin was obtained in MS^2^ mode of the full-scan chromatogram. The accurate masses of TeA, AOH, AME, and TEN were 197.10, 258.05, 272.07, and 414.18, respectively, in the negative mode; the ionized form of *Alternaria* toxins in [M−H]^−^; and the accurate masses of these ionized *Alternaria* toxins were 196.10, 257.05, 271.07, and 413.18, respectively. From the scan filter, each mycotoxin was detected, along with the peak times: TeA, 1.8 min; AOH, 3.98 min; AME, 5.42 min; and TEN, 5.00 min.

The four conjugated *Alternaria* toxins (AOH-3-S, AME-3-S, AOH-9-Glc, and AME-3-Glc) were screened using a Waters Acquity UPLC VION^TM^ ion mobility quadrupole time-of-flight mass spectrometer (Water, Milford, MA, USA), which contained an Acquity UPLC HSS T3 (1.8 μm, 2.1 × 100 mm) column for separation. The column temperature was set at 40 °C. The mobile phase comprised acetonitrile, containing 0.1% (*v*/*v*) formic acid as eluent A, and ultra-pure water, containing 0.1% (*v*/*v*) formic acid, as eluent B. A gradient elution was applied as follows: 5% of mobile phase A was initially used and linearly increased to 80% within 2.5 min and 90% within 2 min and then maintained for 1.5 min. Then, column re-equilibration was performed, resulting in a total run time of 6 min. The flow rate was set to 0.3 mL/min.

The IMS QToF MS analysis for the conjugated toxins was conducted in negative ion mode (ESI^-^) at a capillary voltage of 2.5 kV, a desolvation temperature of 350 °C, a source temperature of 125 °C, a desolvation gas flow of 800 L/h, and a cone nitrogen gas flow of 50 L/h. Argon was used as the collision gas at a pressure of 4 × 10^−3^ mbar. The injection volume was 3 μL. High-definition mass spectrometry (HDMS^E^) was used as the acquisition mode with low- and high-collision energies set to 6 eV and 30–60 eV, respectively. The data were acquired between 50 and 1000 m/z. Lock mass correction was performed by infusing a leucine enkephalin solution (0.2 ng/L) into the ion source every 5 min (*m*/*z* 554.2615 used as the reference ion in the negative mode). Ion mobility and mass calibration were determined using a Major Mix IMS/ToF Calibration Kit (Waters Corporation). The other parameters for data acquisition and processing were set according to the manufacture’s guidelines and performed using UNIFI 1.8.2 software (Waters Corporation).

### 5.9. Statistical Analysis

The results of this study were analyzed with Microsoft Office Excel 2010, and a line chart was plotted using Origin 2018. The data shown in the results are the means of triplicate (or more) values and expressed as ± SD (standard deviation) for the lesion diameter and mycotoxin content. Statistical analysis was performed using SPSS statistical package 18.0. One-way analysis of variance (ANOVA) and Turkey’s HSD test were performed to determine the significance of the main factors and their interactions. *p* < 0.01 was considered statistically significant.

## Data Availability

Not applicable.
